# Lipid composition and cell surface hydrophobicity of *Candida albicans* influence the efficacy of fluconazole–gentamicin treatment

**DOI:** 10.1002/yea.3455

**Published:** 2020-01-10

**Authors:** Jakub Suchodolski, Jakub Muraszko, Aleksandra Korba, Przemysław Bernat, Anna Krasowska

**Affiliations:** ^1^ Department of Biotransformation, Faculty of Biotechnology University of Wrocław Wrocław Poland; ^2^ Department of Industrial Microbiology and Biotechnology, Faculty of Biology and Environmental Protection University of Łódź Łódź Poland

**Keywords:** *Candida albicans*, Cdr1, cell surface hydrophobicity, ergosterol, fluconazole synergism, gentamicin

## Abstract

Adherence of the fungus, *Candida albicans* *,* to biotic (e.g. human tissues) and abiotic (e.g. catheters) surfaces can lead to emergence of opportunistic infections in humans. The process of adhesion and further biofilm development depends, in part, on cell surface hydrophobicity (CSH). In this study, we compared the resistance of *C. albicans* strains with different CSH to the most commonly prescribed antifungal drug, fluconazole, and the newly described synergistic combination, fluconazole and gentamicin. The hydrophobic strain was more resistant to fluconazole due to, among others, overexpression of the *ERG11* gene encoding the fluconazole target protein (CYP51A1, Erg11p), which leads to overproduction of ergosterol in this strain. Additionally, the hydrophobic strain displayed high efflux activity of the multidrug resistance Cdr1 pump due to high expression of the *CDR1* gene. On the other hand, the hydrophobic *C. albicans* strain was more susceptible to fluconazole–gentamicin combination because of its different effect on lipid content in the two strains. The combination resulted in ergosterol depletion with subsequent Cdr1p mislocalization and loss of activity in the hydrophobic strain. We propose that *C. albicans* strains with different CSH may possess altered lipid metabolism and consequently may differ in their response to treatment.

## 1 INTRODUCTION

Lipids, including phospholipids, sphingolipids, and sterols, are crucial constituents of biological membranes in eukaryote cells. Both lipids and lipid‐signalling molecules regulate cell proliferation, viability and, in the case of pathogenic microorganisms, virulence (Pan, Hu, & Yu, 2018). Recently, an increasing number of immunocompromised patients has led to more systemic fungal infections caused by yeast‐like fungi such as *Candida, Cryptococcus*, and *Malassezia* spp. (Pinto, Gonçalves, Cavaleiro, & Salgueiro, 2017; Ricna et al., 2019; Underhill & Pearlman, 2015).

Recognized antifungal targets are the enzymes involved with lipid metabolism within fungi or the lipids themselves (Pan et al., 2018). In clinical situations, the most common antifungal used is fluconazole, which inhibits ergosterol metabolism by targeting cytochrome P‐450 lanosterol 14α‐demethylase (CYP51A1, Erg11p), which is encoded by the *ERG11* gene (Alizadeh, Khodavandi, & Zalakian, 2017). However, fungi are increasingly developing resistance to fluconazole (Beardsley, Halliday, Chen, & Sorrell, 2018; Ortiz & Torres, 2018; Wiederhold, 2017). In the case of the most studied fungus, *Candida albicans* *,* three major resistance mechanisms have been reported. These involve alterations to the target enzyme, either via overexpression or point mutations of the *ERG11* gene (Wu, Gao, Li, Gao, & Ying, 2017) or overproduction of multidrug resistance transporters, which transport fluconazole out of the fungal cell (Paul & Moye‐Rowley, 2014). Cdr1p, belonging to the ATP‐binding cassette family, is the most important of the three identified multidrug resistance transporters involved in fluconazole efflux in *C. albicans* (Prasad, Balzi, Banerjee, & Khandelwal, 2019).

Strategies for overcoming drug resistance in *C. albicans* include either developing new drugs or discovering synergistic combinations of fluconazole with other molecules (Fiori & Van Dijck, 2012; Perlin, 2015).

Recent published works have pointed to a role for the cell wall of *C. albicans* in tolerance to fluconazole (Sorgo et al., 2011). The composition of the cell wall is variable, with cell surface hydrophobicity (CSH; Krasowska & Sigler, 2014) affecting both the adhesion and the pathogenic processes of *C. albicans* (Biniarz, Baranowska, Feder‐Kubis, & Krasowska, 2015; Krasowska & Sigler, 2014). This study shows that both ergosterol metabolism and Cdr1p efflux activity are altered in the hydrophobic *C. albicans* strain, which impacts susceptibility towards fluconazole and the newly described synergistic combination of fluconazole and gentamicin. Based on these results, differences in the physiology of cells, which vary in CSH, should be considered when developing new antifungal therapies.

## 2 MATERIALS AND METHODS

### 2.1 Chemicals

The chemicals and reagents used in this study were purchased from the following sources: gentamicin sulphate (Galfarm; Kraków, Poland); 2‐deoxy‐D‐glucose, fluconazole, hexadecane, rhodamine 6G (R6G), β‐mercaptoethanol (BME), ethylenediaminetetraacetic acid (EDTA), ergosterol, lanosterol, cholesterol (CHOL), N,O‐bis (trimethylsilyl) trifluoroacetamide/trimethylchlorosilane, acetonitrile, isopropanol, formic acid, and ammonium formate (Sigma‐Aldrich; Poznań, Poland); D‐glucose, bacteriological agar, zymolyase, D‐sorbitol, and Tris (manufacturer: Bioshop; distributor: Lab Empire; Rzeszów, Poland); yeast extract (manufacturer: BD; distributor: Diag‐med; Warszawa, Poland); soy peptone (Merck; Warszawa, Poland); chloroform (CHCl_3_) and methanol (MetOH; Chempur; Piekary Śląskie, Poland); KOH and HCl (Avantor; Gliwice Poland); hexane (manufacturer: JT Baker; distributior: Avantor; Gliwice Poland); phospholipids standards: phosphatidic acid (PA 12:0/12:0), phosphatidylcholine (PC 14:0/14:0), phosphatidylethanolamine (PE 14:0/14:0), phosphatidylglycerol (PG 14:0/14:0), phosphatidylserine (PS 14:0/14:0), and phosphatidylinositol (PI 16:0/16:0; Avanti Polar Lipids; Alabama, USA); Ficoll (Pharmacia; Uppsala, Sweden); pyridinium, 4‐(2‐(6‐(dibutylamino)‐2‐naphthalenyl)ethenyl)‐1‐(3‐sulfopropyl) and hydroxide inner salt (di‐4‐ANEPPS; Thermo Fisher; Warszawa, Poland). All chemicals were high purity grade.

### 2.2 Strains and growth conditions

The *C. albicans* strains used in the present study are listed in Table 1. CAF2‐1 and CAF4‐2 were kind gifts from Professor D. Sanglard (Lausanne, Switzerland). KS052, KS063, KS068 and KS069 were constructed during our study. Strains were pregrown at 28°C on yeast extract peptone dextrose (YPD) medium (2% glucose, 1% soy peptone, 1% yeast extract) in a shaking incubator (120 rpm). Agar in a final concentration of 2% was used for medium solidification.

**Table 1:** *Candida albicans* strains used in this study

Strain	Genotype	Reference
CAF2‐1	*ura3Δ::imm434/URA3*	Fonzi and Irwin (1993)
CAF4‐2	*ura3Δ::imm434/ura3Δ::imm434*	Fonzi and Irwin (1993)
KS052	*ura3Δ::imm434/URA3* *CDR1/CDR1‐GFP‐NAT1*	This study
KS063	*ura3Δ::imm434/URA3* *CDR2/CDR2‐GFP‐NAT1*	This study
KS068	*ura3Δ::imm434/ura3Δ::imm434* *CDR1/CDR1‐GFP‐NAT1*	This study
KS069	*ura3Δ::imm434/ura3Δ::imm434* *CDR2/CDR2‐GFP‐NAT1*	This study

For the experiments, cells were grown in 20 ml of YPD medium (28°C; shaking: 120 rpm; starting A_600_ = 0.1; with or without fluconazole, gentamicin, or the combination of both drugs added at *t* = 0 hr) until they reached the stationary phase (24 hr). Cells were then centrifuged (4.5 k rpm, 5 min), washed twice (4.5 k rpm, 5 min) with either phosphate‐buffered saline (PBS) or 50 mM HEPES–NaOH buffer (pH 7.0), and resuspended in either PBS or HEPES–NaOH to the indicated A_600_.

### 2.3 Strain construction

Plasmid p*GFP‐NAT1* (Milne, Cheetham, Lloyd, Aves, & Bates, 2011) was a generous gift from Professor S. Bates (Exeter, United Kingdom). The *CDR1‐GFP‐NAT1* cassette was amplified from pGFP‐NAT1 with the primer pair, C1_GFPNAT_F and C1_GFPNAT_R; *CDR2‐GFP‐NAT1* cassette was amplified from pGFP‐NAT1 with the primer pair, C2_GFPNAT_F and C2_GFPNAT_R (Table 2).

**Table 2:** Primers used in this study

Primer	Sequence 5′ ‐ 3′
C1_GFPNAT_F	CATTCTTACGGTGATCTTTTATTGGTTAGCTAGAGTTCCAAAGGGTAACAGAGAGAAAAAAAATAAGAAAGGTGGTGGTTCTAAAGGTGAAGAATTATT
C1_GFPNAT_R	AACAACAACAATAGTCTAAAAACGTCTATTATATTTTAGACGTTTGAGATACCACCATGTCAAAAAACAACGTTAGTATCGAATCGACAGC
C2_GFPNAT_F	CATTCTTACTATTTTCTTTTACTGGTTGGCTAGAGTTCCAAAAGGTAATAGAGAAAAGAAGATGAAAAAAGGTGGTGGTTCTAAAGGTGAAGAATTATT
C2_GFPNAT_R	ATCAAACAATCACAAATAACGTATAAATAATAATAAGAAAAAAAAAATATGAATACTAATTGTAAAATAACGTTAGTATCGAATCGACAGC
NAT1_F	GCTTATAGATACAGAACTTCTGTTCC
NAT1_R	TGAAACCCATTCTTCTATAAGCATG
C1NAT1_SF	TCAAGCTATGCTTTCTACTGGA
C2NAT1_SF	GTATTGGCTGGTCCTAATGTG
GFP_N1_SR2	AATTCTTCACCTTTAGAACCACC
RDN18F	AGAAACGGCTACCACATCCAA
RDN18R	GGGCCCTGTATCGTTATTTATTGT
ERG11F	TTTGGTGGTGGTAGACATA
ERG11R	GAACTATAATCAGGGTCAGG
CDR1F	TTTAGCCAGAACTTTCACTCATGAT
CDR1R	TATTTATTTCTTCATGTTCATATGGATTGA

*C. albicans* strains were transformed by electroporation with the linear gel‐purified *CDR1‐GFP‐NAT1* or *CDR2‐GFP‐NAT1* cassettes according to the protocol by Sasse et al. (2011). The presence of the *NAT1* marker was verified using the primer pair, NAT1_F and NAT1_R (Table 2). The correct integration of the cassette into the genomic locus was verified using the primer pair, C1NAT1_SF and GFP_N1_SR2 (KS052 and KS068 strains) or C2NAT1_SF and GFP_N1_SR2 (KS063 and KS069 strains; Table 2).

### 2.4 Viability and synergism determination

Experiments were performed according to the Clinical and Laboratory Standards Institute (2008), 3rd ed. M27‐A3 with modifications described before by Suchodolski, Feder‐Kubis, and Krasowska (2017). Briefly, viability was determined by serially diluting fluconazole, gentamicin, or a combination of fluconazole and gentamicin in YPD medium using 96‐well sterile plates (Sarstedt; Stare Babice, Poland) and then inoculated with *C. albicans* suspensions (final A_600_ per well = 0.01). After incubating at 28°C for 24 hr, A_600_ was measured (ASYS UVM 340 Biogenet). The percentage of CAF2‐1 and CAF4‐2 viability was determined by normalizing A_600_ in the control experiments (without antimicrobial agents) as 100%.

### 2.5 Cell surface hydrophobicity

This assay was performed according to Biniarz et al. (2015), with modifications. Briefly, 1 ml of hexadecane was added to the *C. albicans* suspensions (PBS, A_600_ = 0.5, 4 ml). The samples were shaken for 3 min, and the phases were allowed to separate for 45 min. The A_600_ of the aqueous phase was then measured, and CSH was calculated according to Biniarz et al. (2015)**.**

### 2.6 Isolation of plasma membranes

Plasma membranes (PMs) were isolated from suspensions of CAF2‐1 and CAF4‐2 (PBS; concentrated to A_600_ = 20) according to the method reported by Krasowska, Chmielewska, Prescha, Váchová, and Sigler (2002). Briefly, cells were resuspended in lysis medium (1 M sorbitol, 0.1 M EDTA, 1% BME, 3 mg/ml zymolyase) and incubated (37°C; 30 min). Protoplasts were then washed with 1.2 M sorbitol, lysed by ice‐cold H_2_O_dd_ shock, and broken by sonication (5‐sec cycles for 2 min; 4°C) using an ultrasonic processor (SONICS Vibra‐cell VCX 130). Cell lysate was centrifuged (4°C; 10 k rpm; 10 min) to remove unbroken material, and the supernatant was ultracentrifuged (4°C; 100 k rpm; 60 min) using a Micro Ultracentrifuge CS150FNX (Hitachi; Tokyo, Japan). The crude PM pellets were suspended in saline solution with the addition of CHCl_3_–MetOH (1:2, vol/vol). The CHCl_3_ layer was concentrated using nitrogen gas after vigorous stirring at 4°C for 16 hr.

### 2.7 Sterol analysis in PMs

Sterol analysis was performed as described previously (Singh, MacKenzie, Girnun, & Del Poeta, 2017). CHCl_3_–KOH 1 ml (1:1, vol/vol) and CHOL 20 μg were added to the evaporated PM samples and incubated at 23°C for 1 hr. HCl 0.325 ml (1 M) and deionized H_2_O 0.125 ml were then added and centrifuged (5 k rpm). The lower layer was separated into fresh tubes, dried, and then N,O‐bis (trimethylsilyl) trifluoroacetamide/trimethylchlorosilane 0.1 ml was added. The samples were incubated at 85°C for 90 min before adding hexane 50 μl to the tubes for vortexing. The analysis was performed with a gas chromatograph (Agilent 7890) equipped with a column HP 5 MS (30 m × 0.25 mm i.d. × 0.25 mm ft) and a 5975C mass detector. The column was heated at 100°C over 0.5 min; then, the temperature was increased to 240°C at a rate of 25°C min^−1^ and finally to 300°C (for 5 min) at a rate of 3°C min^−1^ with helium gas as a carrier (flow rate = 1 ml min^−1^; Singh et al., 2017). The temperature of the injection port was 250°C. CHOL was used as an internal standard. Trimethylsilyl‐derived ergosterol and lanosterol were analysed according to retention times and fragmentation spectra for standards. Trimethylsilyl ethers of the other sterol metabolites were identified by comparison with the NIST MS database or literature data and quantitated using a standard curve for lanosterol.

### 2.8 Phospholipid analysis in PMs

Phospholipid concentrations were determined using an Agilent 1200 High‐Performance Liquid Chromatography system (Agilent; Santa Clara, USA) and a 4500 Q‐TRAP mass spectrometer (Sciex; Redwood City, USA) equipped with an electrospray ionization (ESI) source. For the chromatographic analysis, 10 μl of the lipid extract (diluted in MetOH–CHCl_3_ in a 4:1 v/v ratio) was injected into a Kinetex C18 column (50 × 2.1 mm, particle size: 5 μm; Phenomenex; Torrance, USA) with a flow rate of 500 μl min^−1^ and a temperature of 40°C. The mobile phases of H_2_O (A) and MetOH (B) included ammonium formate 5 mM. The solvent gradient was initiated at 70% B, increased to 95% B over 1.25 min, and then maintained at 95% B for 6 min before returning to the initial solvent composition over 3 min. The following ion sources for the mass spectrometer settings were applied: curtain gas 25, nebulizer gas 50, turbo gas 60, spray voltage—4.500 V and temperature 600°C. Data analysis was performed with the Analyst™ v1.6.2 software (Sciex; Redwood City, USA).

Phospholipid concentrations were determined qualitatively according to methods described elsewhere (Bernat, Gajewska, Szewczyk, Słaba, & Długoński, 2014). A phospholipid standard for each class of phospholipids was then prepared as PA 12:0/12:0, PC 14:0/14:0, PE 14:0/14:0, PG 14:0/14:0, PS 14:0/14:0, and PI 16:0/16:0 to establish a quantitative method with multiple reaction monitoring transitions.

### 2.9 Isolation of lipid droplets

Lipid droplets (LDs) were isolated by modifying *Schizosaccharomyces pombe* protocol, reported by Mannik, Meyers, and Dalhaimer (2014). Briefly, at least 5 g of CAF2‐1 or CAF4‐2 cells were harvested and resuspended in lysis medium (1 M sorbitol, 0.1 M EDTA, 1% BME, 5 mg zymolyase per gram of wet cells) and incubated (37°C; 60 min; 180 rpm). Protoplasts were then washed with 1.2M sorbitol, resuspended in 12% Ficoll, 10 mM Tris–HCl, and 200μM EDTA, later broken with glass beads (3 × 60 s cycles; 4°C). Cell lysate was overlaid with 12% Ficoll, 10 mM Tris–HCl, and 200μM EDTA buffer and ultracentrifuged (4°C; 100 k rpm; 90 min; rotor deceleration = 0) using a Micro Ultracentrifuge CS150FNX (Hitachi; Tokyo, Japan). Top floating layer containing LDs was transferred to new ultracentrifuge tubes, overlaid one‐third full with 12% Ficoll, 10 mM Tris–HCl, and 200μM EDTA buffer and additionally overlaid two‐third full with 8% Ficoll, 10 mM Tris–HCl, and 200μM EDTA buffer. After ultracentrifugation (4°C; 100 k rpm; 60 min; rotor deceleration = 0), the top floating layer was transferred to new ultracentrifuge tubes, overlaid one‐third full with 600 mM sorbitol, 8% Ficoll, 10 mM Tris–HCl, and 200 μM EDTA buffer and additionally overlaid two‐third full with 250 mM sorbitol, 10 mM Tris–HCl, and 200 μM EDTA buffer. After another ultracentrifugation (4°C; 100 k rpm; 60 min; rotor deceleration = 0), the top layer of purified LDs was harvested. For lipid isolation, a mixture of CHCl_3_–MetOH (1:2, vol/vol) was added, and after vigorous stirring at 4°C for 4 h, the CHCl_3_ layer was concentrated using nitrogen gas.

### 2.10 Sterol and steryl esters analysis in LDs

Lipid samples from LDs were diluted in 1ml CHCl_3_–MetOH (1:4, vol/vol). Then, 10 μl of lipid extract was measured using an Agilent 1200 High‐Performance Liquid Chromatography system and a 4500 Q‐TRAP mass spectrometer equipped with an ESI source and Kinetex C18 column (50 × 2.1 mm, particle size: 5 μm; Phenomenex; Torrance, USA) with a flow rate of 500 μl min^−1^ and a temperature of 40°C. The mobile phases of H_2_O (A) and a mixture of acetonitrile:isopropanol (5:2) included 5 mM ammonium formate and 0.1% formic acid (B). The solvent gradient was initiated at 35% B, increased to 100% B over 4 min; after 11 min, it returned to 35% B over 2 min; the flow rate was set to 0.6 ml min^−1^ (Bernat et al., 2018). Mass spectrometry was recorded under positive mode with enhanced mass spectrum scan type. The following ESI conditions were applied: turbo spray source voltage, 5,500 V; source temperature: 550°C; GS1: 60.00, GS2: 50.00, curtain gas: 25; scan range, 300–900 Da. Ergosterol esters were monitored at m/z 379.3 ([M + H‐fatty acid]^+^) according to the method described by Shui et al. (2010).

### 2.11 Di‐4‐ANEPPS assay

The PM potential (Δψ) of CAF2‐1 and CAF4‐2 was measured using di‐4‐ANEPPS fluorescent dye, according to the protocol of Suchodolski and Krasowska (2019). For data analysis, the red–blue signal ratio was calculated by dividing the sum of fluorescence intensities (IFs) between 580 and 620 nm by the sum of IFs between 540 and 580 nm, as described previously (Suchodolski & Krasowska, 2019).

### 2.12 Real time polymerase chain reaction

RNA was isolated from the CAF2‐1 and CAF4‐2 suspensions (PBS; A_600_ = 10) using the Total RNA Mini Kit (A&A Biotechnology; Gdynia, Poland). Synthesis of cDNA and calculation of gene expression levels were performed according to Szczepaniak, Łukaszewicz, and Krasowska (2015). The following gene‐specific primers were used: RDN18F and RDN18R, ERG11F and ERG11R, and CDR1F and CDR1R (Table 2).

### 2.13 R6G efflux assay

*C. albicans* suspensions (HEPES‐NaOH; A_600_ = 1.0; 25 ml) were treated with 2‐deoxy‐D‐glucose and stained with R6G according to the protocol of Szczepaniak, Cieślik, Romanowicz, Musioł, and Krasowska (2017). In each condition, the R6G uptake was always ≥95%. IFs were collected 15 min after R6G efflux initiation and normalized to 1 for the efflux activity of nontreated CAF2‐1 cells.

### 2.14 Microscopic studies

The strains, KS052 and KS068, were suspended in PBS, concentrated, and observed under a Zeiss Axio Imager A2 microscope equipped with a Zeiss Axiocam 503 mono microscope camera and a Zeiss HBO100 mercury lamp.

### 2.15 Statistical analysis

At least three independent replicates were performed for each experiment. Statistical significance was determined using Student's *t* test (binomial, unpaired).

## 3 RESULTS AND DISCUSSION

### 3.1 *C. albicans* susceptibility to the fluconazole‐gentamicin combination depends on CSH

**Figure 1:**
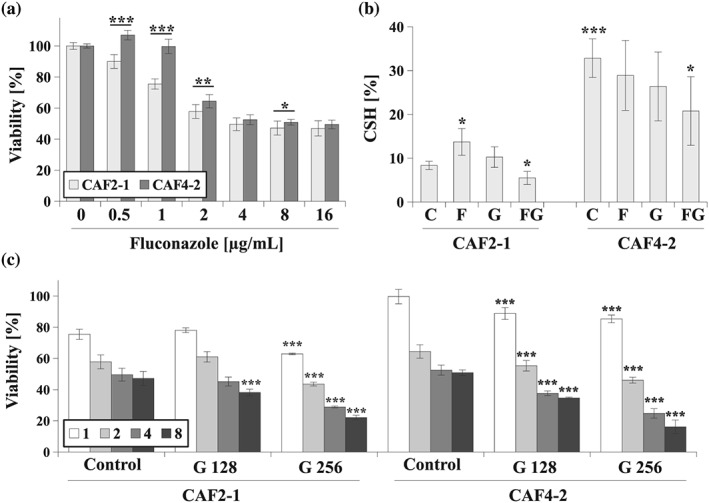
(a) Percentage of viability of the *Candida albicans* CAF2‐1 and CAF4‐2 strains in the presence of fluconazole (0–16 μg/ml) after culture in yeast extract peptone dextrose (YPD) medium for 24 hr (mean ± *SD*, *n* = 6). Statistical analysis was performed by comparing the percentage viability of both strains at the same concentrations; (b) Percentage of cell surface hydrophobicity (CSH, presented as mean ± *SD*, *n* = 3) of the *C. albicans* CAF2‐1 and CAF4‐2 strains grown in the following conditions: C—control without antimicrobial agents, F—treated with fluconazole 4 μg/mL, G—treated with gentamicin 256 μg/ml, FG—simultaneously treated with fluconazole 4 μg/ml and gentamicin 256 μg/ml. Statistical analysis was performed by comparing either untreated CAF4‐2 with untreated CAF2‐1 or treated strains with untreated strains; (c) Percentage of viability of the *C. albicans* CAF2‐1 and CAF4‐2 strains in the presence of fluconazole (1–8 μg/ml, chart legend) and in presence of gentamicin 128 or 256 μg/ml (G 128 and G 256, respectively; mean ± *SD*, *n* = 6). Statistical analysis was performed by comparing viability at the same fluconazole concentrations between samples treated and untreated with gentamicin. Statistical significance in all cases is presented as follows: *p < .05; **p < .01; ***p < .001

CSH is an important feature in the adhesion of pathogenic microorganisms to abiotic and biotic surfaces (Krasowska & Sigler, 2014). In the case of *C. albicans* *,* higher CSH causes greater tolerance towards the antiadhesive properties of biosurfactants (Biniarz et al., 2015). As clinical strains of *Candida* spp. differ in CSH (Silva‐Dias et al., 2015), we aimed to assess the effect of fluconazole with and without gentamicin on *C. albicans* strains with different CSH (Figure 1).

At a fluconazole concentration range of 0.5–2 μg/ml, the hydrophobic *C. albicans* CAF4‐2 strain had increased resistance compared with the hydrophilic CAF2‐1 strain (Figure 1a). At higher fluconazole concentrations, the vulnerability of both strains was similar. This effect might have occurred due to prolonged fluconazole treatment, which can induce a resistant *C. albicans* phenotype (Morschhäuser, 2016). To overcome this effect, we simultaneously tested the effect of the antibacterial drug, gentamicin, and the antifungal drug, fluconazole (Figure 1b–c). We observed that treatment with fluconazole and fluconazole–gentamicin combination had different effects on the hydrophobicity of both strains (Figure 1b). Under control conditions (Figure 1b, trial: C), the CAF4‐2 strain was over threefold more hydrophobic than CAF2‐1, which is similar to what was shown in our previous studies (Biniarz et al., 2015). However, fluconazole and gentamicin both increased the CSH of the hydrophilic CAF2‐1 strain, but reduced CSH in the CAF4‐2 strain (Figure 1b, trial: F or G). In turn, the fluconazole–gentamicin combination reduced CSH in both strains (Figure 1b, trial: FG).

The combination of fluconazole and gentamicin decreased the viability of both *C. albicans* strains (Figure 1c). Further, the combination was more effective against the hydrophobic CAF4‐2 strain. At a gentamicin concentration of 128 μg/ml, there was no difference in susceptibility to fluconazole for the hydrophilic CAF2‐1 strain, whereas for the CAF4‐2 strain, we observed 20% greater viability reduction. At a higher gentamicin concentration of 256 μg/ml, a decrease of viability was present for both strains, but was greater for the CAF4‐2 strain (Figure 1c).

### 3.2 Lipid metabolism is affected differently in hydrophilic and hydrophobic *C. albicans* strains after treatment with the fluconazole–gentamicin combination

**Figure 2:**
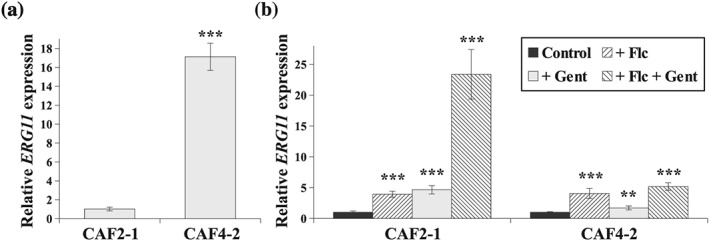
**(**a) Relative *ERG11* gene expression in the *Candida albicans* CAF4–2 strain compared with *C. albicans* CAF2‐1. Statistical analysis was performed by comparing both experiments. (b) Relative *ERG11* gene expression in the *C. albicans* CAF2‐1 and CAF4‐2 strains grown in the following conditions: control without antimicrobial agents, Flc—treated with fluconazole 4 μg/ml, Gent—treated with gentamicin 256 μg/ml, Flc + Gent—simultaneously treated with fluconazole 4 μg/ml and gentamicin 256 μg/ml. Statistical analysis was performed by comparing the *ERG11* expression level of treated with untreated strains, separately. Gene expression levels are reported as mean ± SD of 2^−ΔΔCT^ values (*n* = 6); normalized to 1 for CAF2‐1 in (a) or separately to CAF2‐1 and CAF4‐2 in (b). Statistical significance in all cases is presented as follows: *p < .05; **p < .01; ***p < .001

The fluconazole resistance that is acquired in clinical *C. albicans* isolates by over‐ or down‐expression of the *ERG11* gene leads to alterations in the fungal sterol profile (Alizadeh et al., 2017; Mukherjee, Chandra, Kuhn, & Ghannoum, 2003). In order to understand the different response of the hydrophobic (CAF4‐2) and hydrophilic (CAF2‐1) strains towards the fluconazole–gentamicin combination (Figure 1), we evaluated *ERG11* gene expression (Figure 2) and the sterol profile (Table 3) of both strains treated with fluconazole, gentamicin, and the fluconazole–gentamicin combination.

**Table 3:** Sterols (μg/mg dry mass of isolated plasma membrane lipids, mean ± *SD*, *n* = 3) in *Candida albicans* CAF2‐1 and CAF4‐2 strains grown in the following conditions: control without antimicrobial agents, Flc—treated with fluconazole 4 μg/ml, Gent—treated with gentamicin 256 μg/ml, Flc + Gent—simultaneously treated with fluconazole 4 μg/ml and gentamicin 256 μg/ml

Strain	Condition	Ergosterol	Lanosterol	24‐methyl‐lanosterol	Eburicol
CAF2‐1	Control	3.52 ± 0.1	0.84 ± 0.08	ND	ND
	Flc	ND	13.26 ± 2.59*	13.6 ± 0.78	6.05 ± 0.64
	Gent	31.88 ± 2.45**	31.43 ± 1.34***	11.72 ± 0.27	ND
	Flc + Gent	55.86 ± 0.08***	28.82 ± 0.15***	8.94 ± 1.32	8.13 ± 1.55
CAF4‐2	Control	103.04 ± 6.88**	10.14 ± 0.16***	ND	ND
	Flc	10.44 ± 0.63**	29.51 ± 0.17***	18.74 ± 2.76	ND
	Gent	31.09 ± 4.74***	3.73 ± 0.45***	ND	ND
	Flc + Gent	2.07 ± 0.16***	8.98 ± 0.75*	5.66 ± 0.38	1.32 ± 0.49

*Note.* Statistical analysis was performed by comparing the sterols of untreated CAF4‐2 with untreated CAF2‐1 (included in the CAF4‐2 control row) or by comparing a separately treated strain with an untreated strain (included in Flc, Gent, and Flc + Gent rows).

Abbreviation: ND, not detected.

* *p* < .05.

** *p* < 0.01.

*** *p* < .001.

Under control conditions without antimicrobial agents, *ERG11* gene expression was about 17‐fold higher in the hydrophobic CAF4‐2 strain than in the hydrophilic CAF2‐1 strain (Figure 2a). Here, analyses were performed in the stationary phase of growth for either of the strain. Under those conditions, the *ERG11* gene expression is reduced in CAF2‐1 strain, which results in the lowest ergosterol content in the stationary phase of growth (Suchodolski, Muraszko, Bernat, & Krasowska, 2019). In the hydrophobic strain, the high *ERG11* gene expression resulted, among others, in approximately 29‐fold higher ergosterol concentrations (Table 3, control trials). As the lanosterol concentration was also higher, it can be assumed that, in general, there is a greater ergosterol biosynthesis pathway activity in the hydrophobic CAF4‐2 strain and that this results in higher concentrations of the final product (ergosterol).

To compare the differences in response of both strains to treatment with the antimicrobial agents, we separately calculated 2^–ΔΔCT^ values for *ERG11* expression by normalizing both control conditions (strains untreated) to the value = 1 (Figure 2b). Both the CAF2‐1 and the CAF4‐2 strains responded similarly to fluconazole treatment in terms of *ERG11* gene expression, with both increasing about fourfold. However, Erg11p activity was inhibited by fluconazole in both cases, as indicated by the accumulation of lanosterol and the appearance of the atypical sterol metabolites, 24‐methyl‐lanosterol and eburicol (Table 3). Martel et al. (2010) reported previously that blocking the activity of lanosterol 14α‐demethylase (Erg11p) resulted in accumulation of lanosterol and its methylated derivatives. In the hydrophilic CAF2‐1 strain, despite the increased level of the *ERG11* transcript, synthesis of the pathway product, ergosterol, was fully blocked by fluconazole (Table 3). In the hydrophobic CAF4‐2 strain, Erg11p activity was not fully inhibited by fluconazole due to the residual presence of ergosterol. The higher *ERG11* expression in this strain may be one of the reasons for higher tolerance of CAF4‐2 towards fluconazole (Figure 1a).

Expression of the *ERG11* gene after treatment with gentamicin alone was about fivefold higher for the CAF2‐1 strain (Figure 2b). This resulted in about 10‐fold higher accumulation of ergosterol, a high level of lanosterol and the presence of 24‐methyl‐lanosterol in this strain (Table 3). Prokhorova et al. (2017) reported that aminoglycosides including gentamicin not only target bacterial ribosomes but also interact with eukaryotic 80S ribosomes leading to inhibition of nearly every aspect of protein synthesis, which most likely may include biosynthesis, degradation, and targeting of ergosterol. These findings may indicate that in CAF2‐1, despite higher expression of the *ERG11* gene and a higher level of ergosterol, demethylation of lanosterol is partially inhibited (Table 3). The hydrophobic CAF4‐2 strain responded differently to gentamicin. Despite a twofold higher expression of the *ERG11* gene (Figure 2b), the level of ergosterol was reduced by about 70% compared with the untreated hydrophilic CAF4‐2 strain (Table 3). We did not observe an increase in the concentration of lanosterol or its methylated derivatives (Table 3). This indicates that gentamicin may have inhibited ergosterol biosynthesis in the CAF4‐2 strain but at a different step than where demethylation of lanosterol occurs.

For both strains treated with the fluconazole–gentamicin combination, the presence of lanosterol and methylated lanosterol derivatives indicated partial inhibition of Erg11p activity (Table 3). However, this combination of drugs resulted in much higher expression of *ERG11* in the CAF2‐1 strain (almost 25‐fold higher) than in CAF4‐2 (5‐fold higher; Figure 2b). This in turn resulted in ergosterol overproduction in CAF2‐1. In the CAF4‐2 strain, the investigated combination of antimicrobial agents resulted in ergosterol production that was even less than after treatment with fluconazole alone (Table 3). This may lead to different responses in the two strains to gentamicin alone and could be one of the reasons why CAF4‐2 is more sensitive than CAF2‐1 to the fluconazole–gentamicin combination (Figure 1c).

The proper ratio of sterols to other lipids in PMs is necessary to maintain physiological structure and fluidity of the PM (Simons & Lkonen, 2000). In *Eukaryota*, the overproduction and elevated levels of sterols were reported to exhibit toxic effects towards the cells (Shimada et al., 2019; Tabas, 2002). In yeast cells, excessive ergosterol is either secreted into the extracellular environment or esterified and stored in lipid droplets (LDs) (Hu et al., 2017; Spanova et al., 2012). Interruption with ergosterol biosynthesis by inhibiting squalene synthase was already reported to affect accumulation LDs in *C. albicans* (Ishida et al., 2011). However, the effect of azole drugs on LDs accumulation was only reported for *Leishmania amazonensis* (De Macedo‐Silva, Urbina, De Souza, & Rodrigues, 2013). Here, we identified that either treatment with fluconazole or fluconazole–gentamicin combination lowers the lipid content of LDs more than twofold when compared with untreated cells, regardless of the *C. albicans* strain (Table 4).

**Table 4:** Lipid content (μg/g, isolated lipid droplet lipids per dry cell mass, mean ± *SD*, *n* = 3), ergosterol and steryl esters (10^6^ counts/mg dry mass of isolated LD lipids, mean ± *SD*, *n* = 3) in *Candida albicans* CAF2‐1 and CAF4‐2 strains grown in the following conditions: control without antimicrobial agents, Flc—treated with fluconazole 4 μg/ml, Gent—treated with gentamicin 256 μg/ml, Flc + Gent—simultaneously treated with fluconazole 4 μg/ml and gentamicin 256 μg/ml

Strain	Condition	Lipid content	Ergosterol	Steryl esters
CAF2‐1	Control	0.46 ± 0.08	2.56 ± 0.63	6.88 ± 0.96
	Flc	0.19 ± 0.08*	9.11 ± 0.4***	27.37 ± 2.63**
	Gent	0.29 ± 0.01	1.02 ± 0.11*	3.45 ± 0.31*
	Flc + Gent	0.17 ± 0.08*	9.9 ± 1**	36.8 ± 9.93*
CAF4‐2	Control	0.52 ± 0.11	21.7 ± 1.2*	74.31 ± 3.59**
	Flc	0.24 ± 0.07*	9.5 ± 2.2**	29.72 ± 1.76*
	Gent	0.37 ± 0.06	4.14 ± 0.8**	12.94 ± 0.25*
	Flc + Gent	0.25 ± 0.04*	3.6 ± 1.6***	9.64 ± 5.59***

*Note.* Statistical analysis was performed by comparing the data of untreated CAF4‐2 with untreated CAF2‐1 (included in the CAF4‐2 control row) or by comparing a separately treated strain with an untreated strain (included in Flc, Gent, and Flc + Gent rows).

Abbreviation: ND, not detected.

* *p* < .05.

** *p* < .01.

*** *p* < .001.

In the hydrophobic CAF4‐2 strain, we have observed a similar trend of ergosterol and steryl esters accumulation as in the case of ergosterol in the PM (Table 3). The highest levels of either ergosterol and steryl esters were observed in untreated CAF4‐2 cells and the lowest after treatment with fluconazole–gentamicin combination (Table 4). On the other hand, the hydrophilic CAF2‐1 strain excessively accumulated both ergosterol and steryl esters after fluconazole treatment (Table 4). Kim et al. (2004) reported that a homologue of ergosterol O‐acyltransferase gene, *ARE2*, which controls the storage and decomposition of sterols in lipid droplets, is induced in *C. albicans* treated with ketoconazole. Ergosterol was not detected in the CAF2‐1 PM, treated with fluconazole (Table 3), so it can be speculated that fluconazole impairs ergosterol transport to PM and promotes its deposition in LDs. The opposite situation was observed treating CAF2‐1 cells with gentamicin, where a decrease of ergosterol and steryl esters was observed in LD fraction (Table 4) and an increase in PM (Table 3). Gentamicin was reported to affect lipid homeostasis in vertebrates (Ibraheem et al., 2014; Li, Shih, & Lee, 2013); thus, probably, it impairs LD sterol storage. Fluconazole–gentamicin treatment elevated the levels of both ergosterol and steryl esters in the LD fraction of CAF2‐1, but with similar rate as fluconazole alone (Table 4).

**Figure 3:**
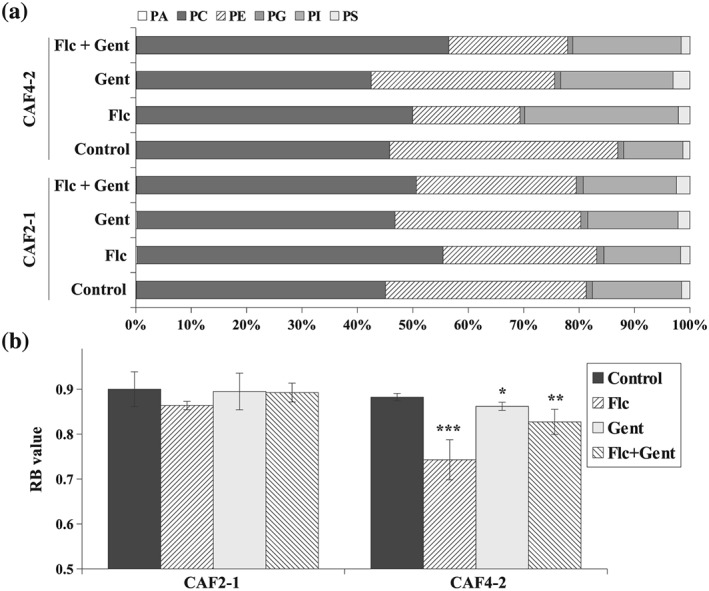
(a) Percentage distribution of phospholipids (phosphatidic acid, PA; phosphatidylcholine, PC; phosphatidylethanolamine, PE; phosphatidylglycerol, PG; phosphatidylinositol, PI and phosphatidylserine, PS) in PMs isolated from CAF2‐1 and CAF4‐2 strains grown in the following conditions: control without antimicrobial agents, Flc—treated with fluconazole 4 μg/ml, Gent—treated with gentamicin 256 μg/ml, Flc + Gent—simultaneously treated with fluconazole 4 μg/ml and gentamicin 256 μg/ml. Included values are means of three independent experiments, *SD* is not shown but was ≤5% in all cases. (b) PM potential (Δψ) expressed as RB values (mean ±*SD*, *n* = 4) calculated from the fluorescence spectra of di‐4‐ANEPPS incorporated into the PMs of the *Candida albicans* CAF2‐1 and CAF4‐2 strains grown in the following conditions: control without antimicrobial agents, Flc—treated with fluconazole 4 μg/ml, Gent—treated with gentamicin 256 μg/ml, Flc + Gent—simultaneously treated with fluconazole 4 μg/ml and gentamicin 256 μg/ml. Statistical analysis was performed by comparing cells treated with antimicrobial agent(s) with the corresponding untreated control (*p < .05; **p < .01; ***p < .001)

Singh, Mahto, and Prasad (2013) reported that fluconazole‐treated *C. albicans* not only alters the sterol profile but also alters the composition of phospholipids (PLs) and sphingolipids, as well as the length and saturation of fatty acids. In PM in vivo, charged PLs are asymmetrically distributed between the two leaflets of the PM, which results in the inner leaflet being negatively charged and a surface potential that binds positively charged ions, proteins, and peptide motifs (Ma, Poole, Goyette, & Gaus, 2017). Among transmembrane transporters (Suchodolski & Krasowska, 2019), these interactions create a transmembrane potential (Δψ; Ma et al., 2017). To check whether the different responses of hydrophobic and hydrophilic strains also depend on lipids other than ergosterol, we evaluated the PL profile of the PMs and the PM potential (Δψ) of both strains treated with fluconazole, gentamicin, and the fluconazole–gentamicin combination (Figure 3).

The untreated CAF4‐2 strain had 5% more PE and 5% less PI than the CAF2‐1 strain (Figure 3a). Only slight differences in composition of PA, PG, or PS were present after treating both strains with the antimicrobial agents. We observed an increase in the PC concentration and a decrease in the PE concentration in the CAF2‐1 strain treated with fluconazole, which is in agreement with previous findings (Singh et al., 2013). However, we saw an approximate 17% increase in the PI concentration in CAF4‐2 treated with fluconazole and about an 11% increase when the strain was treated with the fluconazole–gentamicin combination (Figure 3a). Gentamicin interacts with PM by specific binding with PI and other negatively charged PLs (Kovács et al., 2012; Lesniak, Pecoraro, & Schacht, 2005). Forge, Zajic, Davies, Weiner, and Schacht (1989) reported that membrane disruption by gentamicin is proportional to the PI or PS content in the model membranes (liposomes). We assumed that the accumulation of PI by the CAF4‐2 strain under fluconazole treatment additionally sensitized these cells towards gentamicin.

Accumulation of negatively charged PIs in the PMs of the CAF4‐2 strain treated with fluconazole caused a strong Δψ reduction and PM depolarization (Figure 3b). For the CAF2‐1 strain treated with fluconazole, only a slight depolarization was present. For both strains, treatment with gentamicin alone did not affect Δψ, whereas treatment with the fluconazole–gentamicin combination restored the depolarization caused by fluconazole alone (Figure 3b).

### 3.3 Activity, localization, and expression of the Cdr1 transporter are altered in the hydrophobic strain treated with the fluconazole–gentamicin combination

**Figure 4:**
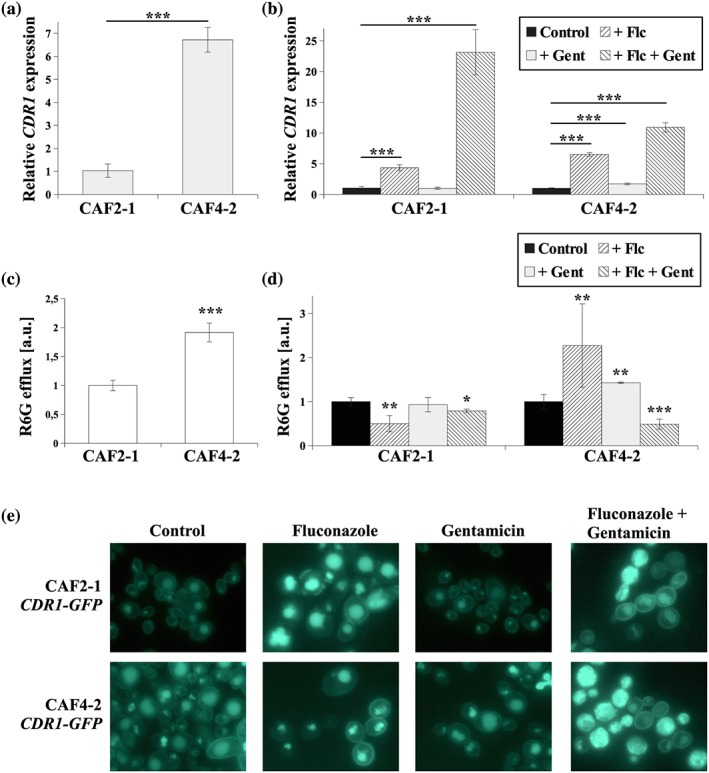
**(**a) Relative *CDR1* gene expression in the *Candida albicans* CAF4‐2 strain compared with the *C. albicans* CAF2‐1 strain. Statistical analysis was performed by comparing both experiments. (b) Relative *CDR1* gene expression in the *C. albicans* CAF2‐1 and CAF4‐2 strains grown in the following conditions: control without antimicrobial agents, Flc—treated with fluconazole 4 μg/ml, Gent—treated with gentamicin 256 μg/ml, Flc + Gent—simultaneously treated with fluconazole 4 μg/ml and gentamicin 256 μg/ml. Statistical analysis was performed by comparing the *CDR1* expression level of treated with untreated strains, separately. Gene expression levels are reported as mean ± *SD* of 2^−ΔΔCT^ values (*n* = 6), normalized to 1 for CAF2‐1 in (a) or separately to CAF2‐1 and CAF4‐2 in (b). (c) Cdr1p‐dependent rhodamine 6G (R6G) efflux in *C. albicans* CAF2‐1 and CAF4‐2 shown as the normalized (normalized to = 1 for CAF2‐1 strain) fluorescence intensity of extracellular R6G (mean ± *SD*, *n* = 6). (d) R6G efflux in the *C. albicans* CAF2‐1 and CAF4‐2 strains grown in the following conditions: control without antimicrobial agents, Flc—treated with fluconazole 4 μg/ml, Gent—treated with gentamicin 256 μg/ml, Flc + Gent—simultaneously treated with fluconazole 4 μg/ml and gentamicin 256 μg/ml (normalized to = 1 separately for untreated CAF2‐1 and CAF4‐2 strains; mean ± *SD*, *n* = 6). (e) Fluorescence micrographs of the subcellular localization of the Cdr1‐GFP protein in the *C. albicans* strains, KS052 (CAF2‐1 CDR1‐GFP) and KS068 (CAF4‐2 CDR1‐GFP) in the following conditions: control without antimicrobial agents, treated with fluconazole 4 μg/ml, treated with gentamicin 256 μg/ml or simultaneously treated with fluconazole 4 μg/ml and gentamicin 256 μg/ml. Scale bar = 5 μm. Statistical significance in all cases is presented as follows: *p < .05; **p < .01; ***p < .001

In *Saccharomyces* *cerevisiae* cells, reduction in Δψ and PM depolarization causes mislocalization of ergosterol and PM proteins from charged membrane domains (Grossmann, Opekarová, Malinsky, Weig‐Meckl, & Tanner, 2007). On the other hand, ergosterol depletion causes mislocalization of *C. albicans*' Cdr1p from PM when expressed in *S. cerevisiae* deficient in ergosterol (Pasrija, Panwar, & Prasad, 2008). The fluconazole–gentamicin combination differentially affected ergosterol content (Table 3) and Δψ (Figure 3b) in hydrophobic and hydrophilic *C. albicans* strains, and so, we checked the effect of this composition on the expression, localization, and activity of Cdr1p in both strains (Figure 4).

The measurements of Cdr1p activity were performed using standard R6G assay (Szczepaniak et al., 2017). R6G dye is, however, a substrate for two paralogous proteins—Cdr1 and Cdr2 (Ivnitski‐Steele et al., 2009). The latter is not constitutively produced by *C. albicans* laboratory strains (Tsao, Rahkhoodaee, & Raymond, 2009), unless exogenous stimulation with xenobiotics, such as fluphenazine (Karababa, Coste, Rognon, Bille, & Sanglard, 2004). To eliminate the role of Cdr2p in herein R6G assay (Figure 4d), we have constructed *CDR2‐GFP* strains in either CAF2‐1 or CAF4‐2 backgrounds (KS063 and KS069, respectively). Neither of the strains displayed Cdr2p‐GFP signal under the control conditions nor treatment with fluconazole, gentamicin, and fluconazole–gentamicin combination (data not shown). Thus, we assumed that R6G efflux was Cdr1‐dependent only (Figure 4d).

In the hydrophobic CAF4‐2 strain, we identified a higher expression of the *CDR1* gene (Figure 4a), higher activity of Cdr1p (Figure 4c), and more intensive fluorescence of Cdr1p‐GFP (Figure 4e) than in the CAF2‐1 strain. Fluconazole induced *CDR1* expression fourfold and almost sevenfold in CAF2‐1 and CAF4‐2, respectively (Figure 4b). However, in the case of CAF2‐1, most of this transporter protein did not localize to the PM. Proper localization was present only in individual cells (Figure 4e, arrow), resulting in low efflux activity (Figure 4d). The reason for this might be total ergosterol depletion under these conditions (Table 3). In contrast, after fluconazole treatment, most CAF4‐2 cells had Cdr1p‐GFP localized to the PMs (Figure 4e) resulting in higher Cdr1p activity (Figure 4d). Fluconazole did not fully deplete ergosterol in CAF4‐2 (Table 3), which allowed Cdr1p to mostly maintain its PM localization and efflux activity.

No differences in *CDR1* gene expression or Cdr1p localization or activity were present after treating CAF2‐1 cells with gentamicin. In the case of the hydrophobic CAF4‐2 strain, higher *CDR1* gene expression (Figure 4b) and transporter activity (Figure 4d) occurred. Treatment with the fluconazole–gentamicin combination resulted in almost 25‐fold higher *CDR1* expression in the CAF2‐1 strain (Figure 4b), leading to higher fluorescence of the Cdr1p‐GFP protein (Figure 4e). Previously, we observed Cdr1p‐GFP mislocalization in stationary phase cells (Szczepaniak et al., 2015), which also occurred here (Figure 4e). However, after treatment with the fluconazole–gentamicin combination, we also noticed dispersion of the protein inside some CAF2‐1 cells. Cdr1p‐GFP localized properly into PMs in most CAF2‐1 cells, most likely because fluconazole–gentamicin treatment leads to overproduction of ergosterol in the CAF2‐1 strain (Table 3), which is crucial for proper Cdr1p localization (Pasrija et al., 2008). The efflux activity of Cdr1p in CAF2‐1 cells treated with the fluconazole–gentamicin combination was similar to the activity in untreated cells. This is probably due to two effects, being delocalization of the transporter in some cells and Cdr1p overproduction (visible as more intense Cdr1p‐GFP fluorescence) in the remainder of the cells (Figure 4).

Fluconazole–gentamicin treatment of *C. albicans* cells leads to ergosterol depletion in the CAF4‐2 strain (Table 3), which resulted in Cdr1p‐GFP dispersion in most of the cells (Figure 4e). Despite higher *CDR1* expression (Figure 4b), the mislocalized protein had reduced efflux activity (Figure 4d). This is another effect that is responsible for the greater inhibitory effect of the fluconazole–gentamicin combination on CAF4‐2 cells (Figure 1c).

## 4 CONCLUSIONS

Differences in the CSH of *C. albicans* may be associated with changes in lipid metabolism and Cdr1 transporter activity and result in resistance to fluconazole or the synergistic combination of fluconazole with other drugs. In our study, the hydrophobic CAF4‐2 strain was more resistant to fluconazole due to ergosterol overproduction and *ERG11* gene overexpression, as well as overproduction and higher activity of the Cdr1 transporter. However, this strain was more susceptible to the synergistic effect of fluconazole with gentamicin, which resulted from substantial ergosterol depletion with treatment, as well as the mislocalization and loss of activity of the Cdr1 efflux pump.

## CONFLICT OF INTEREST

The authors declare no conflict of interest.

## References

[yea3455-bib-0001] Alizadeh, F. , Khodavandi, A. , & Zalakian, S. (2017). Quantitation of ergosterol content and gene expression profile of ERG11 gene in fluconazole‐resistant *Candida albicans* . Current Medical Mycology, 3(1), 13–19. 10.29252/cmm.3.1.13 PMC5747584 29302625

[yea3455-bib-0002] Beardsley, J. , Halliday, C. L. , Chen, S. C. A. , & Sorrell, T. C. (2018). Responding to the emergence of antifungal drug resistance: Perspectives from the bench and the bedside. Future Microbiology, 13(10), 1175–1191. 10.2217/fmb-2018-0059 30113223 PMC6190174

[yea3455-bib-0003] Bernat, P. , Gajewska, E. , Szewczyk, R. , Słaba, M. , & Długoński, J. (2014). Tributyltin (TBT) induces oxidative stress and modifies lipid profile in the filamentous fungus *Cunninghamella elegans* . Environmental Science and Pollution Research, 21(6), 4228–4235. 10.1007/s11356-013-2375-5 24306727 PMC3945233

[yea3455-bib-0004] Bernat, P. , Nykiel‐Szymańska, J. , Stolarek, P. , Słaba, M. , Szewczyk, R. , & Różalska, S. (2018). 2,4‐dichlorophenoxyacetic acid‐induced oxidative stress: Metabolome and membrane modifications in *Umbelopsis isabellina*, a herbicide degrader. PLoS ONE, 13(6), e0199677 10.1371/journal.pone.0199677 29933393 PMC6014680

[yea3455-bib-0005] Biniarz, P. , Baranowska, G. , Feder‐Kubis, J. , & Krasowska, A. (2015). The lipopeptides pseudofactin II and surfactin effectively decrease *Candida albicans* adhesion and hydrophobicity. Antonie Van Leeuwenhoek. International Journal of General and Molecular Microbiology, 108(2), 343–353. 10.1007/s10482-015-0486-3 PMC4491367 26021480

[yea3455-bib-0006] Clinical and Laboratory Standards Institute (Ed.) (2008). Reference method for broth dilutionantifungal susceptibility testing of yeast. In: Approved standard. M27‐A3 28 (3rd ed.). Wayne, PA: Clinical and Laboratory Standards Institute.

[yea3455-bib-0007] De Macedo‐Silva, S. T. , Urbina, J. A. , De Souza, W. , & Rodrigues, J. C. F. (2013). In vitro activity of the antifungal azoles itraconazole and posaconazole against *Leishmania amazonensis* . PLoS ONE, 8(12), e83247 10.1371/journal.pone.0083247 24376670 PMC3871555

[yea3455-bib-0008] Fiori, A. , & Van Dijck, P. (2012). Potent synergistic effect of doxycycline with fluconazole against *Candida albicans* is mediated by interference with iron homeostasis. Antimicrobial Agents and Chemotheraphy, 56(7), 3785–3796. https://doi, 10.1128/AAC.06017-11 PMC3393430 22564841

[yea3455-bib-0009] Fonzi, W. A. , & Irwin, M. Y. (1993). Isogenic strain construction and gene mapping in *Candida albicans* . Genetics, 134, 717–728. 8349105 10.1093/genetics/134.3.717 PMC1205510

[yea3455-bib-0010] Forge, A. , Zajic, G. , Davies, S. , Weiner, N. , & Schacht, J. (1989). Gentamicin alters membrane structure as shown by freeze‐fracture of liposomes. Hearing Research, 37, 129–139. 10.1016/0378-5955(89)90035‐X. 2536649

[yea3455-bib-0011] Grossmann, G. , Opekarová, M. , Malinsky, J. , Weig‐Meckl, I. , & Tanner, W. (2007). Membrane potential governs lateral segregation of plasma membrane proteins and lipids in yeast. EMBO Journal, 26, 1–8. 10.1038/sj.emboj.7601466 17170709 PMC1782361

[yea3455-bib-0012] Hu, Z. , He, B. , Ma, L. , Sun, Y. , Niu, Y. , & Zeng, B. (2017). Recent advances in ergosterol biosynthesis and regulation mechanisms in *Saccharomyces cerevisiae* . Indian Journal of Microbiology, 57(3), 270–277. 10.1007/s12088-017-0657-1 28904410 PMC5574775

[yea3455-bib-0013] Ibraheem, Z. O. , Basir, R. , Aljobory, A. K. , Ibrahim, O. E. , Alsumaidaee, A. , & Yam, M. F. (2014). Impact of gentamicin coadministration along with high fructose feeding on progression of renal failure and metabolic syndrome in sprague‐dawley rats. BioMed Research International, 2014, article number: 823879 10.1155/2014/823879. 25045706 PMC4090614

[yea3455-bib-0014] Ishida, K. , Fernandes Rodrigues, J. C. , Cammerer, S. , Urbina, J. A. , Gilbert, I. , de Souza, W. , & Rozental, S. (2011). Synthetic arylquinuclidine derivatives exhibit antifungal activity against *Candida albicans*, *Candida tropicalis* and *Candida parapsilopsis* . Annals of Clinical Microbiology and Antimicrobials, 10(3), 1–10. 10.1186/1476-0711-10-3 21255433 PMC3036746

[yea3455-bib-0015] Ivnitski‐Steele, I. , Holmes, A. R. , Lamping, E. , Monk, B. C. , Cannon, R. D. , & Sklar, L. A. (2009). Identification of Nile Red as a fluorescent substrate of the *Candida albicans* ABC transporters Cdr1p and Cdr2p and the MFS transporter Mdr1p. Analytical Biochemistry, 394(1), 87–91. 10.1016/j.ab.2009.07.001 19577533 PMC2739806

[yea3455-bib-0016] Karababa, M. , Coste, A. T. , Rognon, B. , Bille, J. , & Sanglard, D. (2004). Comparison of gene expression profiles of *Candida albicans* azole‐resistant clinical isolates and laboratory strains exposed to drugs inducing multidrug transporters. Antimicrobial Agents and Chemotherapy, 48(8), 3064–3079. 10.1128/AAC.48.8.3064-3079.2004 15273122 PMC478486

[yea3455-bib-0017] Kim, K. Y. , Shin, Y. K. , Park, J. C. , Kim, J. H. , Yang, H. , Han, D. M. , & Paik, Y. K. (2004). Molecular cloning and biochemical characterization of *Candida albicans* acyl‐CoA:sterol acyltransferase, a potential target of antifungal agents. Biochemical and Biophysical Research Communications, 319(3), 911–919. 10.1016/j.bbrc.2004.05.076 15184069

[yea3455-bib-0018] Kovács, E. , Savopol, T. , Iordache, M. M. , Sǎplǎcan, L. , Sobaru, I. , Istrate, C. , … Moisescu, M. G. (2012). Interaction of gentamicin polycation with model and cell membranes. Bioelectrochemistry, 87, 230–235. 10.1016/j.bioelechem.2012.03.001 22522030

[yea3455-bib-0019] Krasowska, A. , Chmielewska, L. , Prescha, A. , Váchová, L. , & Sigler, K. (2002). Viability and formation of conjugated dienes in plasma membrane lipids of *Saccharomyces cerevisiae*, *Schizosaccharomyces pombe*, *Rhodotorula glutinis* and *Candida albicans* exposed to hydrophilic, amphiphilic and hydrophobic pro‐oxidants. Folia Microbiologica, 47, 145–151. 10.1007/BF02817672 12058392

[yea3455-bib-0020] Krasowska, A. , & Sigler, K. (2014). How microorganisms use hydrophobicity and what does this mean for human needs? Frontiers in Cellular and Infection Microbiology, 4, 112 10.3389/fcimb.2014.00112 25191645 PMC4137226

[yea3455-bib-0021] Lesniak, W. , Pecoraro, V. L. , & Schacht, J. (2005). Ternary complexes of gentamicin with iron and lipid catalyze formation of reactive oxygen species. Chemical Research in Toxicology, 18, 357–364. 10.1021/tx0496946 15720143

[yea3455-bib-0022] Li, Y. C. , Shih, Y. M. , & Lee, J. A. (2013). Gentamicin caused renal injury deeply related to methylglyoxal and Neε‐(carboxyethyl)lysine (CEL). Toxicology Letters, 219(1), 85–92. 10.1016/j.toxlet.2013.01.024 23454834

[yea3455-bib-0023] Ma, Y. , Poole, K. , Goyette, J. , & Gaus, K. (2017). Introducing membrane charge and membrane potential to T cell signaling. Frontiers in Immunology, 8, 1513 10.3389/fimmu.2017.01513 29170669 PMC5684113

[yea3455-bib-0024] Mannik, J. , Meyers, A. , & Dalhaimer, P. (2014). Isolation of cellular lipid droplets: Two purification techniques starting from yeast cells and human placentas. Journal of Visualized Experiments, 86, e50981 10.3791/50981 PMC4160924 24747783

[yea3455-bib-0025] Martel, C. M. , Parker, J. E. , Bader, O. , Weig, M. , Gross, U. , Warrilow, A. G. S. , … Kelly, S. L. (2010). A clinical isolate of *Candida albicans* with mutations in ERG11 (encoding sterol 14α‐demethylase) and ERG5 (encoding C22 desaturase) is cross resistant to azoles and amphotericin B. Antimicrobial Agents and Chemotherapy, 54(9), 3578–3583. 10.1128/AAC.00303-10 20547793 PMC2934972

[yea3455-bib-0026] Milne, S. W. , Cheetham, J. , Lloyd, D. , Aves, S. , & Bates, S. (2011). Cassettes for PCR‐mediated gene tagging in *Candida albicans* utilizing nourseothricin resistance. Yeast, 28, 833–841. 10.1002/yea.1910 22072586

[yea3455-bib-0027] Morschhäuser, J. (2016). The development of fluconazole resistance in *Candida albicans*—An example of microevolution of a fungal pathogen. Journal of Microbiology, 54(3), 192–201. 10.1007/s12275-016-5628-4 26920879

[yea3455-bib-0028] Mukherjee, P. K. , Chandra, J. , Kuhn, D. M. , & Ghannoum, M. A. (2003). Mechanism of fluconazole resistance in *Candida albicans* biofilms: Phase‐specific role of efflux pumps and membrane sterols. Infection and Immunity, 71(8), 4333–4340. 10.1128/IAI.71.8.4333-4340.2003 12874310 PMC165995

[yea3455-bib-0029] Ortiz, C. , & Torres, R. (2018). Antifungal resistance and its evolution: An increasing concern. Advances in Biotechnology & Microbiology, 10(1), 555777 10.19080/aibm.2018.10.555777

[yea3455-bib-0030] Pan, J. , Hu, C. , & Yu, J.‐H. (2018). Lipid biosynthesis as an antifungal target. Journal of Fungi, 4, articel number: 50 10.3390/jof4020072. PMC6023442 29677130

[yea3455-bib-0031] Pasrija, R. , Panwar, S. L. , & Prasad, R. (2008). Multidrug transporters CaCdr1p and CaMdr1p of *Candida albicans* display different lipid specificities: Both ergosterol and sphingolipids are essential for targeting of CaCdr1p to membrane rafts. Antimicrobial Agents and Chemotherapy, 52(2), 694–704. 10.1128/AAC.00861-07 18056285 PMC2224756

[yea3455-bib-0032] Paul, S. , & Moye‐Rowley, W. S. (2014). Multidrug resistance in fungi: Regulation of transporter‐encoding gene expression. Frontiers in Physiology, 5, 143 10.3389/fphys.2014.00143 24795641 PMC3997011

[yea3455-bib-0033] Perlin, D. S. (2015). Echinocandin Resistance in *Candida* . Clinical Infectious Diseases, 61(S6), S612–S617. 10.1093/cid/civ791 26567278 PMC4643482

[yea3455-bib-0034] Pinto, E. , Gonçalves, M.‐J. , Cavaleiro, C. , & Salgueiro, L. (2017). Antifungal activity of *Thapsia villosa* essential oil against *Candida*, *Cryptococcus*, *Malassezia*, *Aspergillus* and dermatophyte species. Molecules, 22, 1595 10.3390/molecules22101595 PMC6151651 28937623

[yea3455-bib-0035] Prasad, R. , Balzi, E. , Banerjee, A. , & Khandelwal, N. K. (2019). All about CDR transporters: Past, present, and future. Yeast, 36(4), 223–233. 10.1002/yea.3356 30192990

[yea3455-bib-0036] Prokhorova, I. , Altman, R. B. , Djumagulov, M. , Shrestha, J. P. , Urzhumtsev, A. , Ferguson, A. , … Yusupova, G. (2017). Aminoglycoside interactions and impacts on the eukaryotic ribosome. Proceedings of the National Academy of Sciences, 114(51), E10899–E10908. 10.1073/pnas.1715501114 PMC5754804 29208708

[yea3455-bib-0037] Ricna, D. , Lengerova, M. , Bezdicek, M. , Kocmanova, I. , Drgona, L. , Weinbergerova, B. , … Racil, Z. (2019). Detection and identification of fungi in bronchoalveolar lavage fluid from immunocompromised patients using panfungal PCR. Folia Microbiologica, 64, 421–428. 10.1007/s12223-018-00669-w 30535753

[yea3455-bib-0038] Sasse, C. , Schillig, R. , Dierolf, F. , Weyler, M. , Schneider, S. , Mogavero, S. , … Morschhäuser, J. (2011). The transcription factor Ndt80 does not contribute to Mrr1‐, Tac1‐, andUpc2‐mediated fluconazole resistance in *Candida albicans* . Plos One, 6, e25623 10.1371/journal.pone.0025623 21980509 PMC3181345

[yea3455-bib-0039] Shimada, T. L. , Shimada, T. , Okazaki, Y. , Higashi, Y. , Saito, K. , Kuwata, K. , … Hara‐Nishimura, I. (2019). High sterol ester 1 is a key factor in plant sterol homeostasis. Nature Plants, 5(11), 1154–1166. 10.1038/s41477-019-0537-2 31712757

[yea3455-bib-0040] Shui, G. , Guan, X. L. , Low, C. P. , Chua, G. H. , Goh, J. S. Y. , Yang, H. , & Wenk, M. R. (2010). Toward one step analysis of cellular lipidomes using liquid chromatography coupled with mass spectrometry: Application to *Saccharomyces cerevisiae* and *Schizosaccharomyces pombe* lipidomics. Molecular BioSystems, 6(6), 1008–1017. 10.1039/b913353d 20485745

[yea3455-bib-0041] Silva‐Dias, A. , Miranda, I. M. , Branco, J. , Monteiro‐Soares, M. , Pina‐Vaz, C. , & Rodrigues, A. G. (2015). Adhesion, biofilm formation, cell surface hydrophobicity, and antifungal planktonic susceptibility: Relationship among *Candida* spp. Frontiers in Microbiology, 6, 205 10.3389/fmicb.2015.00205 25814989 PMC4357307

[yea3455-bib-0042] Simons, K. , & Lkonen, E. (2000). How cells handle cholesterol. Science, 290(5497), 1721–1726. 10.1126/science.290.5497.1721 11099405

[yea3455-bib-0043] Singh, A. , MacKenzie, A. , Girnun, G. , & Del Poeta, M. (2017). Analysis of sphingolipids, sterols, and phospholipids in human pathogenic *Cryptococcu*s strains. The Journal of Lipid Research, 58, 2017–2036. 10.1194/jlr.M078600 28811322 PMC5625125

[yea3455-bib-0044] Singh, A. , Mahto, K. K. , & Prasad, R. (2013). Lipidomics and in vitro azole resistance in *Candida albicans* . OMICS: A Journal of Integrative Biology, 17(2), 84–93. 10.1089/omi.2012.0075 23374108 PMC3567621

[yea3455-bib-0045] Sorgo, A. G. , Heilmann, C. J. , Dekker, H. L. , Bekker, M. , Brul, S. , de Koster, C. G. , … Klis, F. M. (2011). Effects of fluconazole on the secretome, the wall proteome, and wall integrity of the clinical fungus *Candida albicans* . Eukaryotic Cell, 10(8), 1071–1081. 10.1128/EC.05011-11 21622905 PMC3165447

[yea3455-bib-0046] Spanova, M. , Zweytick, D. , Lohner, K. , Klug, L. , Leitner, E. , Hermetter, A. , & Daum, G. (2012). Influence of squalene on lipid particle/droplet and membrane organization in the yeast *Saccharomyces cerevisiae* . Biochimica et Biophysica Acta ‐ Molecular and Cell Biology of Lipids, 1821(4), 647–653. 10.1016/j.bbalip.2012.01.015 PMC3790963 22342273

[yea3455-bib-0047] Suchodolski, J. , Feder‐Kubis, J. , & Krasowska, A. (2017). Antifungal activity of ionic liquids based on (−)‐menthol: A mechanism study. Microbiological Research, 197, 56–64. 10.1016/j.micres.2016.12.008 28219526

[yea3455-bib-0048] Suchodolski, J. , & Krasowska, A. (2019). Plasma membrane potential of *Candida albicans* measured by di‐4‐ANEPPS fluorescence depends on growth phase and regulatory factors. Microorganisms, 7(4), 110 10.3390/microorganisms7040110 PMC6518178 31022974

[yea3455-bib-0049] Suchodolski, J. , Muraszko, J. , Bernat, P. , & Krasowska, A. (2019). A crucial role for ergosterol in plasma membrane composition, localisation, and activity of Cdr1p and H + ‐ATPase in *Candida albicans* . Microorganisms, 7(10), 378 10.3390/microorganisms7100378 PMC6843828 31546699

[yea3455-bib-0050] Szczepaniak, J. , Cieślik, W. , Romanowicz, A. , Musioł, R. , & Krasowska, A. (2017). Blocking and dislocation of *Candida albicans* Cdr1p transporter by styrylquinolines. International Journal of Antimicrobial Agents, 50, 171–176. 10.1016/j.ijantimicag.2017.01.044 28602766

[yea3455-bib-0051] Szczepaniak, J. , Łukaszewicz, M. , & Krasowska, A. (2015). Estimation of *Candida albicans* ABC transporter behavior in real‐time via fluorescence. Frontiers in Microbiology, 6, 1382 10.3389/fmicb.2015.01382 26696990 PMC4673308

[yea3455-bib-0052] Tabas, I. (2002). Consequences of cellular cholesterol accumulation: Basic concepts and physiological implications. Journal of Clinical Investigation, 110(7), 905–911. 10.1172/JCI200216452 12370266 PMC151158

[yea3455-bib-0053] Tsao, S. , Rahkhoodaee, F. , & Raymond, M. (2009). Relative contributions of the *Candida albicans* ABC transporters Cdrlp and Cdr2p to clinical azole resistance. Antimicrobial Agents and Chemotherapy, 53(4), 1344–1352. 10.1128/AAC.00926-08 19223631 PMC2663127

[yea3455-bib-0054] Underhill, D. M. , & Pearlman, E. (2015). Immune interactions with pathogenic and commensal fungi: a two‐way street. Immunity, 43, 845–858. 10.1016/j.immuni.2015.10.023 26588778 PMC4865256

[yea3455-bib-0055] Wiederhold, N. P. (2017). Antifungal resistance: Current trends and future strategies to combat. Infection and Drug Resistance, 10, 249–259. 10.2147/IDR.S124918 28919789 PMC5587015

[yea3455-bib-0056] Wu, Y. Q. , Gao, N. , Li, C. , Gao, J. , & Ying, C. M. (2017). A newly identified amino acid substitution T123I in the 14α‐demethylase (Erg11p) of *Candida albicans* confers azole resistance. FEMS Yeast Research, 17(3), 1–6. 10.1093/femsyr/fox012 28334124

